# Obinutuzumab after anti-rituximab antibody-associated treatment failure in membranous nephropathy: a case report

**DOI:** 10.3389/fimmu.2026.1758250

**Published:** 2026-04-13

**Authors:** Jianying Guo, Anni Zhong, Di Wu, Yang Liu, Yuna Chen, Yi Xu, Ricong Xu, Qijun Wan

**Affiliations:** Department of Nephrology, Shenzhen Second People’s Hospital, First Affiliated Hospital of Shenzhen University, Shenzhen, Guangdong, China

**Keywords:** anti-PLA2R antibodies, anti-rituximab antibodies (ARA), membranous nephropathy, obinutuzumab, rituximab resistance

## Abstract

Rituximab is an established therapy for primary membranous nephropathy, but anti−rituximab antibodies (ARA) have been associated with rituximab treatment failure. We report a 66−year−old woman with PLA2R−positive membranous nephropathy who developed ARA positivity and clinical features consistent with rituximab treatment failure, including B−cell reconstitution, undetectable serum rituximab levels, and worsening proteinuria despite declining anti−PLA2R antibody titers. Kidney biopsy confirmed stage III membranous nephropathy. She was subsequently treated with the humanized type II anti−CD20 antibody obinutuzumab, which induced complete depletion of circulating B cells, achieved therapeutic serum concentration, follow by both immunological and clinical remission within 8.5 months, despite persistent ARA positivity. This case suggests that obinutuzumab may be an effective rescue option after rituximab failure associated with ARA and underscores the importance of integrating therapeutic drug monitoring, ARA detection, and B−cell kinetics assessment in the management of refractory membranous nephropathy.

## Introduction

Membranous nephropathy (MN) represents the leading cause of nephrotic syndrome in adults, accounting for 20-37% of primary nephrotic syndrome cases (1). Anti-phospholipase A2 receptor (PLA2R) antibodies are detectable in 70-80% of patients with primary MN and correlate with disease activity (2). Rituximab (RTX), a chimeric type I anti-CD20 monoclonal antibody, is recommended as a first-line immunosuppressive option for moderate- to high-risk primary MN according to current guidelines, achieving complete or partial remission in approximately 60% of patients at 24 months in the MENTOR trial (3, 4). However, primary non-response or relapse occurs in a substantial proportion of patients, necessitating alternative therapeutic strategies.

The development of anti-rituximab antibodies (ARA) has emerged as an important mechanism of treatment failure, occurring in 23-47% of MN patients receiving RTX (5, 6). These antibodies can potentially interfere with drug efficacy through multiple mechanisms, including accelerated drug clearance and interference with CD20 binding, leading to subtherapeutic drug concentrations and poor clinical outcomes (5). Obinutuzumab (OBI), a glycoengineered type II humanized anti-CD20 antibody, offers theoretical advantages including enhanced B-cell cytotoxicity through increased antibody-dependent cellular cytotoxicity (ADCC) and potentially reduced immunogenicity due to its humanized structure (7, 8).

While preliminary reports suggest OBI may be effective in RTX-refractory MN, systematic evaluation of immunological parameters, including ARA titers and drug concentrations, has been limited (9, 10). We present longitudinal monitoring data from a patient with PLA2R-associated MN who developed ARA-associated treatment failure and was subsequently treated with OBI, permitting integrated assessment of drug exposure, anti-drug antibody dynamics, and clinical response. These findings provide descriptive, hypothesis-generating insights into treatment monitoring in this clinical context.

## Case presentation

A 66-year-old woman (BMI 20.83 kg/m²) with controlled hypertension and hyperlipidemia presented with lower extremity edema and foamy urine, and had no family history of kidney disease. Initial laboratory evaluation showed serum creatinine 53 umol/L, urine protein-to-creatinine ratio (UPCR) 4.28 g/g, serum albumin 36.1 g/L, and anti-PLA2R antibody titer 28.06 RU/mL (reference <14 RU/mL). Kidney biopsy was recommended but declined by the patient. She was initially managed conservatively with losartan potassium 25–50 mg daily and atorvastatin 20 mg daily.

After 7 months of conservative management, disease progression was evident with anti-PLA2R antibodies rising to 224.2 RU/mL, albumin decreasing to 29.4 g/L, and persistent nephrotic-range proteinuria (UPCR 4.32 g/g). The patient again declined kidney biopsy but consented to immunosuppressive therapy. Rituximab was initiated at 1 g intravenously on days 1 and 15.

### Initial response and subsequent failure

Two weeks following RTX administration (at month 7.5), an encouraging initial response was observed: anti-PLA2R antibodies decreased to 175 RU/mL, and circulating CD19+ B cells were effectively depleted from 9.8% to 0.2%. At month 10 (3 months after RTX), anti-PLA2R antibodies remained noticeably decreased to 35.85 RU/mL, but proteinuria showed no evident improvement. However, at month 14 (7 months after RTX), anti-PLA2R antibodies had rebounded to 62.48 RU/mL and B-cell reconstitution occurred (CD19+ 12.9%). ARA were first detected at this time point (29.62 IU/mL; reference ≤24 IU/mL), and serum RTX concentration was below the assay’s lower limit of quantification (<0.5 ug/mL).

Given these findings suggesting inadequate B-cell suppression, a single RTX intensification dose (1 g) was administered. RTX intensification was well tolerated, and no infusion-related hypersensitivity reactions or anaphylaxis occurred despite the presence of ARA (29.62 IU/mL). Vital signs remained stable during infusion, and no delayed hypersensitivity reactions were observed during follow-up. At month 15.5 (1.5 months after RTX intensification), the anti-PLA2R antibody decreased to 17.72 RU/mL, and B cells were nearly cleared again (CD19+ 0.3%), but proteinuria remained elevated. Subsequently, at month 19 (5 months after intensification), clinical deterioration was evident: anti-PLA2R antibodies rebounded again to 30.56 RU/mL, proteinuria increased to 7.10 g/d (24-h urinary protein) and serum albumin decreased to 22.1 g/L. ARA titers had risen to 60.82 IU/mL, and RTX remained undetectable.

### Kidney biopsy and treatment switch

**Figure 1:**
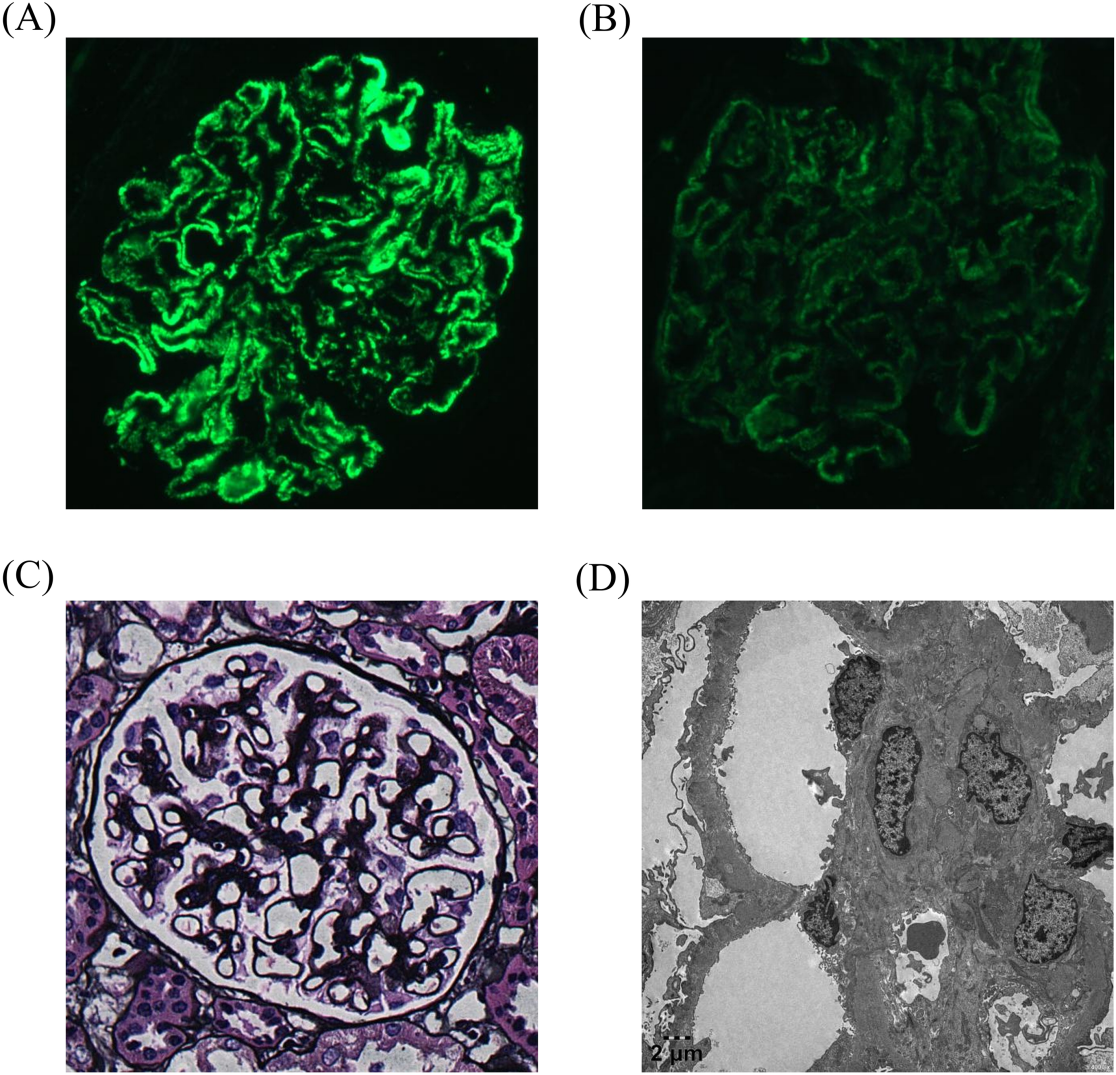
Renal biopsy findings. **(A)** Immunofluorescence microscopy demonstrating strong granular deposits of IgG4 (+++) along the glomerular capillary loops in a diffuse pattern. **(B)** Positive immunofluorescence staining for PLA2R antigen (+) along the glomerular basement membrane, confirming PLA2R-associated membranous nephropathy. **(C)** Light microscopy with PASM staining (×400) showing diffusely thickened glomerular capillary walls with spike-like projections, open but rigid-appearing capillary lumens, and mild diffuse mesangial cell and matrix proliferation. **(D)** Electron microscopy revealing irregularly thickened glomerular basement membrane (approximately 1000nm) with electron-dense deposits in subepithelial locations and within the basement membrane. Some deposits appear encircled by basement membrane material and show absorption features. Podocyte foot processes show diffuse effacement. These findings are consistent with stage III membranous nephropathy.

At this juncture, with clear evidence of treatment failure, the patient consented to kidney biopsy. Histopathological examination revealed stage III membranous nephropathy with extensive subepithelial electron-dense deposits, diffuse podocyte foot process effacement (>80%), and granular PLA2R staining along glomerular capillary walls (**Figure 1**). Immunofluorescence demonstrated IgG4-dominant deposition (3+ intensity) in a granular capillary wall pattern.

In the setting of ARA positivity and RTX treatment failure, therapy was switched to obinutuzumab 1 g intravenously on days 1 and 15. At the time of switching, ARA remained positive (most recent titer 60.82 IU/mL). During the first OBI infusion, the patient experienced infusion reaction (chills, low-grade fever 38.3 °C, tachycardia) at 1 hour, managed successfully with temporary infusion interruption, intravenous dexamethasone 10 mg, and rate reduction upon resumption.

### Response to obinutuzumab

**Figure 2:**
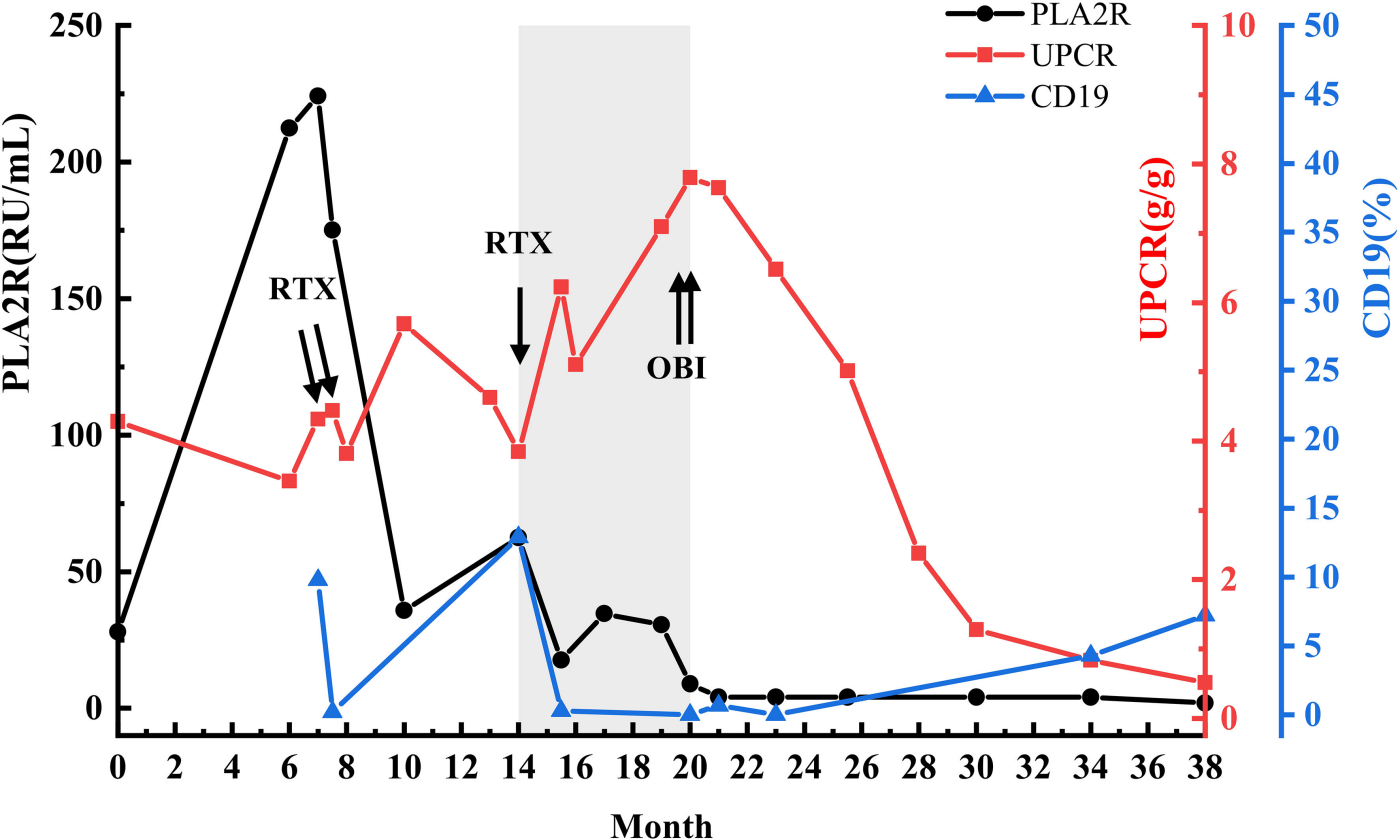
Temporal relationships between immunological markers and clinical parameters during anti-CD20 therapy. The grey-shaded area highlights the period during which anti-rituximab antibodies (ARA) were detectable, illustrating the dissociation between declining anti-PLA2R antibodies and worsening proteinuria despite ongoing rituximab (RTX) therapy. Dynamic changes in anti-PLA2R antibodies (black line, left y-axis), UPCR (red line, right y-axis), and circulating CD19+ B cells (blue line, right y-axis) over the treatment course. Arrows indicate the timing of RTX and obinutuzumab (OBI) administrations. This figure uses a unified timescale with **Table 1** and the main text to facilitate cross-reference.

**Figure 3:**
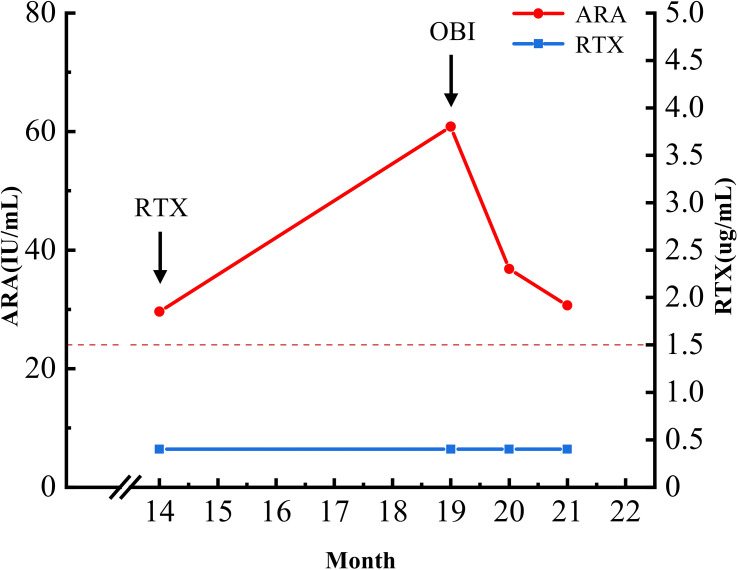
Temporal relationship between anti-rituximab antibody (ARA) titers and drug concentrations during treatment course. Serial assessments during months 14–21 captured rising ARA titers and undetectable rituximab (RTX) levels, coinciding with the observed dissociation between declining anti-PLA2R antibodies and worsening proteinuria. Further measurements were not performed following the reduction of proteinuria and achievement of clinical remission. ARA levels (red line, left y-axis) and RTX concentrations (blue line, right y-axis) are shown over time. Arrows indicate RTX and OBI administration. The dashed line indicates the assay cutoff for ARA positivity.

OBI therapy resulted in a rapid immunological response: anti-PLA2R antibodies became undetectable (<5 RU/mL) within 1.5 months (at month 20), accompanied by near-complete B-cell depletion (CD19+ 0.69%) (**Figure 2**). Importantly, OBI concentration at month 21 (1.5 months after OBI) was 452.08 ug/mL, well above reported therapeutic thresholds at the measured time point, despite persistent ARA (30.67 IU/mL), suggesting minimal immunological cross-reactivity (**Table 1**, **Figure 3**). Subsequently, at month 23 (3.5 months after OBI), B cells remained fully depleted (CD19+ 0%), and anti-PLA2R antibodies remained undetectable.

**Table 1:** Laboratory parameters at key time points from disease onset.

Time	Creatinine (umol/L)	Proteinuria (24-h or UPCR)	Albumin (g/L)	Anti-PLA2R (RU/mL)	CD19 (%)	ARA (IU/mL)	Drug level (ug/mL)	Treatment
0 mo	53	4.28	36.1	28.06	–	–	–	Supportive therapy
6 mo	49.6	3.42	25.9	212.33	–	–	–	–
7 mo	49.9	4.32	29.4	224.2	9.8	–	–	RTX
7.5 mo	53.7	4.44^*^	28.1	175	0.2	–	–	RTX
8 mo	–	3.82	–	–	–	–	–	–
10 mo	58.3	5.69	35.4	35.85	–	–	–	–
13 mo	89.7	4.63	30.9	–	–	–	–	–
14 mo	67.4	3.85^*^	23.4	62.48	12.9	ARA: 29.62	RTX<0.5	RTX
15.5 mo	75.2	6.23	29.4	17.72	0.3	–	–	–
16 mo	–	5.11	–	–	–	–	–	–
17 mo	70.6	–	27.4	34.66	–	–	–	–
19 mo	72.8	7.1^*^	22.1	30.56	–	ARA: 60.82	RTX<0.5	OBI (19.5 mo)
20 mo	72.5	7.8	25.3	8.95	0	ARA: 36.83	RTX<0.5	OBI
21 mo	73.3	7.66	34.8	<5	0.69	ARA: 30.67	RTX<0.5 OBI: 452.08	–
23 mo	67.1	6.48	35.2	<5	0	–	–	–
25.5 mo	68.6	5.01	38.3	<5	–	–	–	–
28 mo	–	2.38	–	–	–	–	–	–
30 mo	63.8	1.28	41.2	<5	–	–	–	–
34 mo	61.1	0.83	43.9	<5	4.3	–	OBI<0.5	–
38 mo	62	0.51	43.5	1.88	7.23	–	OBI<0.5	–

ARA, anti-rituximab antibody; OBI, obinutuzumab; RTX, rituximab; UPCR, urine protein-to-creatinine ratio; wk, week; mo, month.

Time is expressed as months (mo) from disease onset and corresponds to the timeline shown in **Figure 2**.

*Values marked with an asterisk represent 24-hour urinary protein excretion (g/d); all other values are expressed as UPCR (g/g) obtained from spot urine samples.

Clinical remission followed the immunological response: partial remission was achieved at month 28 (8.5 months after OBI) (UPCR 2.38 g/g, albumin 38.3 g/L), with continued improvement at month 38 (18.5 months after OBI) (UPCR 0.51 g/g, albumin 43.5 g/L). Anti-PLA2R antibodies were undetectable and renal function remained stable throughout. However, B-cell reconstitution was detected at month 34 (CD19+ 4.3%), with a further rise through month 38 (CD19+ 7.23%). (**Table 1**, **Figure 2**).

## Discussion

This case provides detailed longitudinal data consistent with anti-rituximab antibody–associated treatment failure in MN and subsequent clinical and immunological remission following obinutuzumab therapy. Our systematic monitoring approach captured the temporal relationship between ARA emergence, loss of drug exposure, and clinical deterioration, while also revealing important dissociations between traditional biomarkers (e.g., anti-PLA2R antibodies) and clinical outcomes.

### Mechanisms of treatment failure

Clinically, ARA positivity has been associated with inferior outcomes, including lower remission rates at 6 and 12 months (31% vs. 56% and 54% vs. 87%, respectively) and higher relapse rates (63% vs. 29%) compared to ARA-negative patients (6). Our patient’s clinical trajectory was broadly consistent with these observations, with ARA emergence occurring after B-cell reconstitution and progressive proteinuria despite RTX intensification.

The development of ARA has been associated with high baseline anti-PLA2R antibody titers and other markers of immunologically active disease, as well as higher body weight or overweight status (11). More broadly, the immunogenicity of therapeutic monoclonal antibodies can be influenced by multiple host and treatment-related factors, including age, sex, genetic background, immune status, concomitant medications, and treatment regimen characteristics (12). In our patient, the anti-PLA2R antibody level was markedly elevated immediately before RTX initiation (224.2 RU/mL), whereas BMI was within the normal range (20.83 kg/m^2^). Although the elevated anti-PLA2R level is consistent with previously reported associations with ARA development, the single-case nature of this report precludes inference regarding causality or individual risk factors.

The mechanisms by which ARA compromise RTX efficacy remain incompletely characterized but may include accelerated drug clearance through immune complex formation, interference with CD20 binding, and functional neutralization of complement-dependent cytotoxicity (CDC) and antibody-dependent cellular cytotoxicity (ADCC) (5). In our case undetectable RTX concentrations (<0.5 ug/mL) in the presence of rising ARA titers may suggest accelerated drug clearance as a potential contributing mechanism, consistent with previous studies showing that RTX concentrations <2 ug/mL predict treatment failure (13).

Notably, at month 14 (7 months after the initial RTX), B-cell reconstitution together with low or undetectable rituximab levels was also compatible with the expected pharmacokinetic waning of rituximab (14). Combined with the borderline-positive ARA at this time point (29.62 IU/mL), these findings suggest that the observed changes were not attributable to ARA alone. Accordingly, the borderline ARA result was not initially interpreted as definitive evidence of ARA-mediated treatment failure, and an RTX intensification dose was administered. The inference of ARA-associated failure instead based on subsequent follow-up after RTX intensification, which demonstrated a rebound in anti-PLA2R antibody levels after an initial decline, persistently high-grade proteinuria, a further increase in ARA titers (29.62 to 60.82 IU/mL), and persistently undetectable rituximab concentrations.

However, low or undetectable RTX levels are not specific to ARA-mediated clearance and may arise from other mechanisms, including inadequate drug exposure, urinary loss in heavy proteinuria and target-mediated clearance via CD20-positive B-cell binding, internalization, and intracellular degradation (15). Urinary RTX was not measured in this case, representing an important limitation. Future studies incorporating urinary RTX quantification and pharmacokinetic sampling could help better clarify the relative contributions of these mechanisms.

### Discordance between biomarkers and clinical outcomes

A particularly instructive aspect of this case is the dissociation between anti-PLA2R antibody titers and clinical parameters following ARA development. While anti-PLA2R antibodies showed an overall downward trend, decreasing from 224.2 to 30.56 RU/mL during RTX therapy, proteinuria paradoxically increased from 4.32 g/g (UPCR) to 7.10 g/d (24-h urinary protein). Previous studies have shown that immunological and clinical responses in membranous nephropathy may be temporally discordant, with reductions in anti-PLA2R antibody levels often preceding decreases in proteinuria by several months (16, 17). Therefore, declining anti−PLA2R titers do not necessarily translate into immediate improvement in proteinuria. However, delayed clinical response alone may not fully account for our patient’s clinical course.

An alternative and biologically plausible explanation is early immunologic reactivation or relapse. Notably, anti-PLA2R titers exhibited two rebounds (from 35.85 RU/mL to 62.48 RU/mL, and from 17.72 RU/mL to 34.66 RU/mL), which temporally coincided with clinical deterioration. During this period, RTX concentrations were undetectable and were accompanied by rising ARA titers (29.62 to 60.82 IU/mL) together with B-cell reconstitution, suggesting inadequate drug exposure and loss of sustained immunologic control, potentially facilitate disease reactivation. In addition, the presence of stage III pathology may have contributed to slower structural recovery, even when immunologic activity was improving. Taken together, rather than attributing the findings solely to “discordance“, we interpret the worsening proteinuria as likely reflecting a combination of delayed clinical response, early immunologic reactivation or relapse, inadequate RTX exposure in the setting of ARA, and chronic structural injury. Owing to the infrequent assessment of anti-PLA2R levels and proteinuria, we cannot determine the precise temporal sequence or infer causality.

Although anti-PLA2R antibody levels are generally useful for monitoring immunologic activity and predicting treatment response in membranous nephropathy, they may not fully reflect disease status in complex therapeutic contexts such as inadequate drug exposure or the presence of anti-drug antibodies. Recent analyses from the MENTOR trial cohort further support that anti-PLA2R antibody levels alone are less informative than when interpreted alongside clinical parameters (18). Accordingly, treatment monitoring in such settings may benefit from an integrated evaluation of drug exposure, anti-drug antibodies, B-cell kinetics, proteinuria, and overall clinical status. As this observation is derived from a single case, further studies are needed to determine how frequently such biomarker discordance occurs and how best to integrate these parameters into clinical decision-making.

### Obinutuzumab as rescue therapy

The favorable clinical course observed after obinutuzumab therapy may reflect differences in pharmacologic and immunologic properties compared with rituximab, although causal inferences cannot be established from a single case. Its humanized structure may reduce immunogenicity compared to chimeric antibodies, and Fc glycoengineering enhances its ADCC activity relative to RTX (7). Critically, only approximately 20% of ARA have been reported to cross-react with OBI due to absence of chimeric epitopes (5). Our monitoring data are consistent with this limited cross-reactivity, as reflected by therapeutic OBI concentrations (452.08 ug/mL) despite persistent ARA.

A recent report suggested that remission rates may reach approximately 80% with OBI in RTX-refractory MN (10). Our case adds valuable pharmacological context to these clinical observations by demonstrating that therapeutic drug exposure may still be achieved despite the presence of ARA.

Safety considerations warrant mention. Despite ARA positivity, RTX re-dosing was well tolerated without infusion-related hypersensitivity. In contrast, an infusion reaction occurred during the first obinutuzumab infusion but resolved with standard supportive measures. This isolated observation does not establish a causal relationship between ARA and infusion reactions.

### Monitoring implications

This case illustrates the potential value of integrated monitoring incorporating ARA detection, drug concentration measurement, and conventional immunological markers. We propose that the combination of ARA positivity, undetectable drug concentrations, and B-cell reconstitution in a patient with persisted proteinuria, even in the setting of declining anti-PLA2R antibodies, may prompt consideration of alternative anti-CD20 agents rather than RTX retreatment. Early recognition of these patterns may prevent futile RTX intensification and enable timely therapeutic adjustment.

### Limitations

Several limitations merit consideration. First, ARA levels were not measured prior to the initial RTX treatment; therefore, pre-existing antibodies cannot be excluded, and the temporal relationship between rituximab exposure and ARA development cannot be clearly established. Second, the single-case nature of this report precludes causal inference or the derivation of treatment algorithms. While the observed associations are consistent with ARA-mediated resistance, no functional experiments were performed to assess ARA neutralizing activity or immunologic cross-reactivity with OBI. Accordingly, whether the efficacy of OBI is related to limited cross-reactivity remains an indirect inference. Third, serial pharmacologic and immunologic assessments were incomplete. OBI concentrations were measured at a single time point and ARA titers were not remeasured during later follow-up. Fourth, the observed response after obinutuzumab may partly reflect cumulative or delayed immunosuppressive effects of prior rituximab exposure, which cannot be fully disentangled in a single-patient observation. Finally, as with all single-case reports, publication bias toward favorable outcomes cannot be excluded, and the generalizability of these findings remains limited.

## Conclusion

This case describes clinical and immunological remission following obinutuzumab therapy in a patient with anti-rituximab antibodies (ARA)-associated treatment failure. The persistence of ARA despite adequate obinutuzumab exposure is consistent with limited cross-reactivity rather than reversal of resistance mechanisms. These descriptive, hypothesis-generating observations highlight the potential value of integrated monitoring of drug exposure and anti-drug antibodies and warrant further study in larger cohorts with functional assays.

## Data Availability

The original contributions presented in the study are included in the article/**Supplementary Material**. Further inquiries can be directed to the corresponding authors.
